# The Effects of Beverage Intake after Exhaustive Exercise on Organ Damage, Inflammation and Oxidative Stress in Healthy Males

**DOI:** 10.3390/antiox10060866

**Published:** 2021-05-28

**Authors:** Takaki Tominaga, Tsukasa Ikemura, Koichi Yada, Kazue Kanda, Kaoru Sugama, Sihui Ma, Wonjun Choi, Mayu Araya, Jiapeng Huang, Nobuhiro Nakamura, Katsuhiko Suzuki

**Affiliations:** 1 Graduate School of Sport Sciences, Waseda University, Tokorozawa 359-1192, Japan; takaki.k-bbc@akane.waseda.jp (T.T.); wonwon11110@fuji.waseda.jp (W.C.); mayu-sum@toki.waseda.jp (M.A.); hjpshidsg1234@toki.waseda.jp (J.H.); 2 Research Fellow of Japan Society for the Promotion of Sciences, Tokyo 102-0083, Japan; masihui@toki.waseda.jp; 3 Faculty of Sport Sciences, Waseda University, Tokorozawa 359-1192, Japan; ikemura@kitasato-u.ac.jp (T.I.); yada_koich@yahoo.co.jp (K.Y.); nnakamura@aoni.waseda.jp (N.N.); 4 Future Innovation Institute, Waseda University, Shinjuku 162-0041, Japan; rurishijimikanda@gmail.com (K.K.); k.sugama@kurenai.waseda.jp (K.S.)

**Keywords:** acute exercise, organ damage, inflammation, oxidative stress, beverage intake, endotoxin

## Abstract

Strenuous exercise induces organ damage, inflammation and oxidative stress. To prevent exercise-induced organ damage, inflammation and oxidative stress, rehydrating may be an effective strategy. In the present study, we aimed to examine whether beverage intake after exhaustive exercise to recover from dehydration prevents such disorders. Thirteen male volunteers performed incremental cycling exercise until exhaustion. Immediately after exercise, the subjects drank an electrolyte containing water (rehydrate trial: REH) or did not drink any beverage (control trial: CON). Blood samples were collected before (Pre), immediately (Post), 1 h and 2 h after exercise. Urine samples were also collected before (Pre) and 2 h after exercise. We measured biomarkers of organ damage, inflammation and oxidative stress in blood and urine. Biomarkers of muscle, renal and intestinal damage and inflammation increased in the blood and urine after exercise. However, changes in biomarkers of organ damage and inflammation did not differ between trials (*p* > 0.05). The biomarker of oxidative stress, thiobarbituric acid reactive substances (TBARS), in plasma, showed different changes between trials (*p* = 0.027). One hour after exercise, plasma TBARS concentration in REH had a higher trend than that in CON (*p* = 0.052), but there were no significant differences between Pre and the other time points in each trial. These results suggest that beverage intake after exercise does not attenuate exercise-induced organ damage, inflammation or oxidative stress in healthy males. However, rehydration restores exercise-induced oxidative stress more quickly.

## 1. Introduction

Strenuous exercise induces not only muscle damage, but also internal organ damage [1,2,3]. Muscle damage decreases muscle strength [4], and intestinal damage results in gastrointestinal bleeding, diarrhea and abdominal pain [5,6]. Severe renal damage also results in acute kidney injury (AKI) [7]. Strenuous exercise perturbs the immune system’s homeostasis through conditions such as leukocytosis, hypercytokinemia, systemic inflammatory response and immune suppression [1,2,3,5,8,9]. Furthermore, severe systemic inflammatory response is one of the pathologies of heat stroke [10,11]. Increased oxidative stress is also a major characteristic of strenuous exercise [1], and severe oxidative stress results in cellular/tissue damage [12]. Therefore, it is crucial to prevent exercise-induced organ damage, inflammation and oxidative stress.

Numerous approaches to prevent exercise-induced organ damage, inflammation and oxidative stress (e.g., pre-exercise cooling, nutritional interventions and other approaches) have been investigated [1]. Among them, rehydrating via beverage intake may be a particularly effective strategy against such disorders. For example, glucose or polyphenol/phytochemical-containing beverage intake attenuates exercise-induced organ damage, inflammation and oxidative stress [13,14,15,16,17,18,19,20]. Furthermore, several studies suggest that hydration only to prevent dehydration may also attenuate such disorders [21,22]. These previous studies have examined the effects of beverage intake before and during exercise. However, few studies have focused on the effects of beverage intake after exercise to recover from dehydration on exercise-induced organ damage, inflammation and oxidative stress.

Although beverage intake before exercise attenuates exercise-induced organ damage and inflammation [21,22], the mechanisms of these preventing effects are not clear. Gut-derived endotoxins are one of the candidates of a trigger of exercise-induced inflammation [1,5]. Strenuous exercise induces gastrointestinal ischemia, intestinal damage and hyperpermeability of the intestine, which results in the leakage of endotoxins [1,5]. Therefore, post-exercise beverage intake may attenuate exercise-induced inflammation via gut-derived endotoxins.

Therefore, we hypothesized that rehydration after exercise attenuates exercise-induced endotoxemia, and as a result, rehydration attenuates exercise-induced organ damage, inflammation and oxidative stress. The purpose of this study is to investigate whether beverage intake after incremental exercise to recover from dehydration prevents organ damage, inflammation and oxidative stress via endotoxemia as a trigger of inflammation in healthy males.

## 2. Materials and Methods

### 2.1. Subjects

This study is an extension of a previously published investigation, utilizing the same participants as described below [23]. This study included thirteen healthy males (age, 22 ± 4 years (mean ± SD); height, 175 ± 6 cm; body mass, 68 ± 9 kg). None of the subjects had documented autonomic dysfunction, cardiovascular or ocular disorders, or took any medications. The Academic Research Ethics Committee of Waseda University approved this protocol, which was carried out in compliance with the Declaration of Helsinki. Before the research began, all subjects signed declarations of informed consent. Before the experiments, the researchers familiarized the experimental protocol to each subject.

### 2.2. Exercise Protocol

As an extension of our previous study, the exercise protocol in this study is also the same [23]. Briefly, the subjects were not allowed to drink caffeinated beverages or perform strenuous exercise for 6 h, nor eat for at least 2 h before the experiment. On arrival at the laboratory, the subjects were instructed to empty their bladder, and their body mass was measured. After a 10 min rest period, the subjects started cycling at 40 W, which was gradually raised by 20 W every 3 min until the subjects could no longer sustain a 60 rpm pedaling cadence. Immediately after the exhaustive exercise, subjects drank 500 mL of electrolyte-containing isotonic water (OS-1, Otsuka Pharmaceutical Factory Inc., Tokyo, Japan) (rehydrate trial: REH) or rested for 1 min without drinking (control trial: CON). The order of trials was randomized, and subjects completed both the REH and CON protocols 1 week apart. Both protocols were carried out under controlled temperatures and humidity (REH: 23.0 ± 0.1 °C, 49.9 ± 0.1% relative humidity (rh), CON: 23.1 ± 0.1 °C, 49.8 ± 0.2% rh).

### 2.3. Blood and Urine Sampling

Blood samples were obtained before (Pre), immediately (Post), 1 h and 2 h after the exercise from the right brachial vein using vacutainers containing no additive or ethylenediaminetetraacetic acid (EDTA)-Na_2_ to obtain serum and plasma samples, respectively. The serum separation vacutainers were left to clot at room temperature for 30 min and then centrifuged at 1400× *g* for 10 min. Vacutainers containing EDTA for plasma separation were immediately centrifuged at 1400× *g* for 10 min. The percentage change in plasma volume was calculated from hemoglobin and hematocrit in the blood [24]. Hemoglobin levels, hematocrit levels and the number of total leukocytes in the blood were determined in EDTA-treated venous whole blood samples using an automatic blood cell counter (pocH-100i, Sysmex Co., Kobe, Japan). Serum and plasma samples were divided into small aliquots, stored at −80 °C for later analyses, and freeze-rethaw cycles were avoided.

The urine samples were obtained before (Pre) and 2 h after exercise. The subjects were instructed to empty their bladder 2 h before Pre sampling. The collected samples were centrifuged at 1400× *g* for 10 min and sediments were removed. The supernatants were also divided into small aliquots, stored at −80 °C for later analyses, and freeze-rethaw cycles were avoided.

### 2.4. Assays for Biochemistry, Organ Damage Markers, Inflammatory Mediators and Oxidative Stress

The concentrations of creatinine, blood urea nitrogen (BUN), derivatives of reactive oxygen metabolites (d-ROMs), biological antioxidant potential (BAP), cystatin-C, myoglobin, insulin, growth hormone, glucose, free fatty acid (FFA), uric acid, urine protein, albumin, *N*-acetyl-β-d-glucosaminidase (NAG) and osmolality were measured by Koutou-Biken Co. (Tsukuba, Japan). Plasma and urinary tumor necrosis factor (TNF)-α, IL-6 and granulocyte colony-stimulating factor (G-CSF) concentrations were measured using a Quantikine high-sensitivity enzyme-linked immunosorbent assay (ELISA) kit (R&D Systems, Minneapolis, MN, USA). Plasma and urinary IL-1 receptor antagonist (IL-1ra), monocyte chemoattractant protein (MCP)-1, macrophage colony-stimulating factor (M-CSF), retinol binding protein 4 (RBP4), IL-18 and IL-18 binding protein α (IL-18BPa) concentrations were measured using a Quantikine ELISA kit (R&D Systems, Minneapolis, MN, USA). Plasma and urinary intestine-fatty acid binding protein (I-FABP) concentrations were measured using a Duoset ELISA kit (R&D Systems, Minneapolis, MN, USA). Plasma and urinary IL-2, IL-4, IL-10 and complement (C) 5a concentrations were measured using an OptEIA ELISA Kit (Beckton Dickinson Biosciences, San Diego, CA, USA). Plasma and urinary myeloperoxidase (MPO) and calprotectin concentrations and plasma lipopolysaccharide binding protein (LBP) concentrations were measured using ELISA kits from Hycult Biotech (Uden, The Netherlands). Plasma neutrophil gelatinase-associated lipocalin (NGAL) concentration was measured using a Quantikine ELISA kit (R&D Systems, Minneapolis, MN, USA), and urinary NGAL concentration was measured using BioPorto^®^ NGAL ELISA Kits (Enzo Life Sciences, Farmingdale, NY, USA). Plasma and urinary ileal-bile acid binding protein (I-BABP) concentrations were measured using a FABP6 ELISA kit (BioVendor Laboratory Medicine, Brno, Czech Republic). Plasma and urinary kidney injury molecule 1 (KIM-1) and cortisol concentrations were measured using an ELISA kit (Enzo Life Sciences). Plasma and urinary liver-fatty acid binding protein (L-FABP) were measured using a Human FABP1 Wide-range ELISA Kit (Uscn Life Science, Wuhan, China). Plasma aldosterone concentration was measured using an ELISA kit (Enzo Life sciences, Farmingdale, NY, USA), and urinary aldosterone concentration was measured using an Aldosterone Parameter Assay Kit (R&D Systems, Minneapolis, MN, USA). Plasma and urinary nitrotyrosine concentrations were measured using an ELISA Kit (StressMarq Biosciences, Victoria, BC, USA). Urinary 8-hydroxy-2′-deoxyguanosine (8-OHdG) concentration was measured using an ELISA Kit (StressMarq Biosciences, Victoria, BC, USA). Plasma endotoxin concentration was measured via PYROGENT™-5000 Kinetic Turbidimetric LAL Assay test (LONZA, Walkersville, MD, USA). When we measured endotoxins, we used endotoxin-free tubes, tips, reservoirs and microplates. The absorbance was measured spectrophotometrically on a VersaMax Microplate Reader (Molecular Devices Inc., San Jose, CA, USA) or Spectra Max iD5 (Molecular Devices Inc., San Jose, CA, USA). Plasma and urinary thiobarbituric acid reactive substances (TBARS) concentrations were measured fluorescently using a TBARS Assay Kit (Cayman Chemical Co., Ann Arbor, MI, USA). The fluorescence was measured on a FLUOstar Optima plate reader (BMG Labtech Ltd., Ortenberg, Germany). The concentrations of each parameter were calculated by comparison with the standard curve established in the same measurement.

Plasma and serum parameters (except serum osmolality) were adjusted according to the percentage change in plasma volume calculated from hemoglobin and hematocrit levels [24]. Urinary parameters were adjusted as the gross amount per minute (raw concentration × urine volume/time) to correct urine condensation as previously described [7].

### 2.5. Statistics

The sample size was calculated using the program G*power [25]. Thirteen subjects were required to detect an effect size of *f* = 0.34 for the within-between interaction, with a power of 0.8 and a significance level of 0.05 under the assumption of a correlation coefficient among repeated measures *r* = 0.5, and a nonsphericity correction of *ε* = 1. The data are shown as mean ± standard error (SE). The Shapiro-Wilk test was used to determine the normality of the data distribution. Prior to analysis, non-normally distributed data were log-transformed. Two-way repeated-measures analysis of variance (ANOVA) was used to analyze the data. When significant time effects were evident, a Bonferroni post hoc test was used to identify the significant differences among mean values. When a significant time × trial interaction was evident, a paired *t*-test with Holm correction was used to identify the significant differences. These statistical analyses were performed with SPSS version 26.0 (IBM Corp., Armonk, NY, USA). Statistical significance was defined as *p* < 0.05.

Because we could not obtain enough plasma samples from some subjects to measure all parameters, the sample size of the measurement of plasma G-CSF, C5a, KIM-1 was *n* = 11, *n* = 11 and *n* = 9, respectively.

## 3. Results

### 3.1. Exercise Duration and Hydration Status

**Figure 1:**
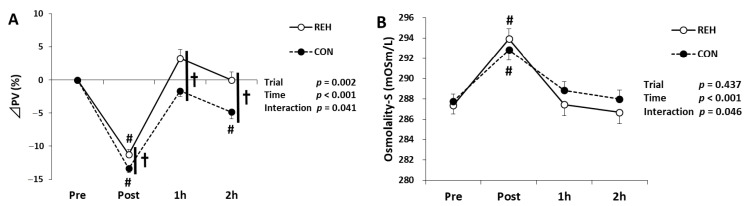
Changes in (**A**) ΔPV (remade based on Ikemura et al. [23]) and (**B**) serum osmolality. Values are presented as mean ± SE. # *p* < 0.05 vs. Pre in each trial. † *p* < 0.05 REH vs. CON in each time point. PV, plasma volume; REH, rehydrate trial; CON, control trial.

There was no difference in the exercise duration to exhaustion in either trial (REH: 26.8 ± 2.0, CON: 27.2 ± 2.0 min, *p* = 0.1) [23]. To investigate the effects of beverage intake on dehydration, we measured the percentage change of plasma volume (ΔPV) and serum osmolality. In CON, ΔPV decreased immediately after exercise in both trials (Figure 1A) [23]. In CON, ΔPV remained lower at the end of the trial, whereas REH did not change (Figure 1A) [23]. Serum osmolality increased immediately after exercise and showed different changes between trials (interaction; *p* = 0.046). However, no significant difference was observed between trials at either time point (Figure 1B). These results indicate that beverage intake after exercise restored exercise-induced dehydration.

### 3.2. Exercise-Induced Organ Damage

**Figure 2:**
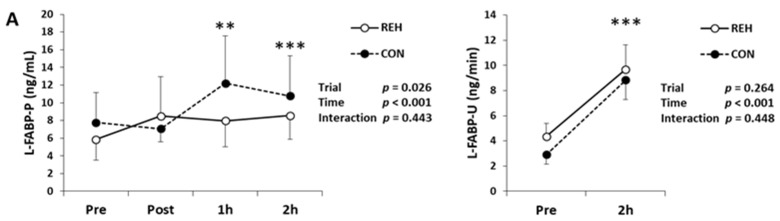

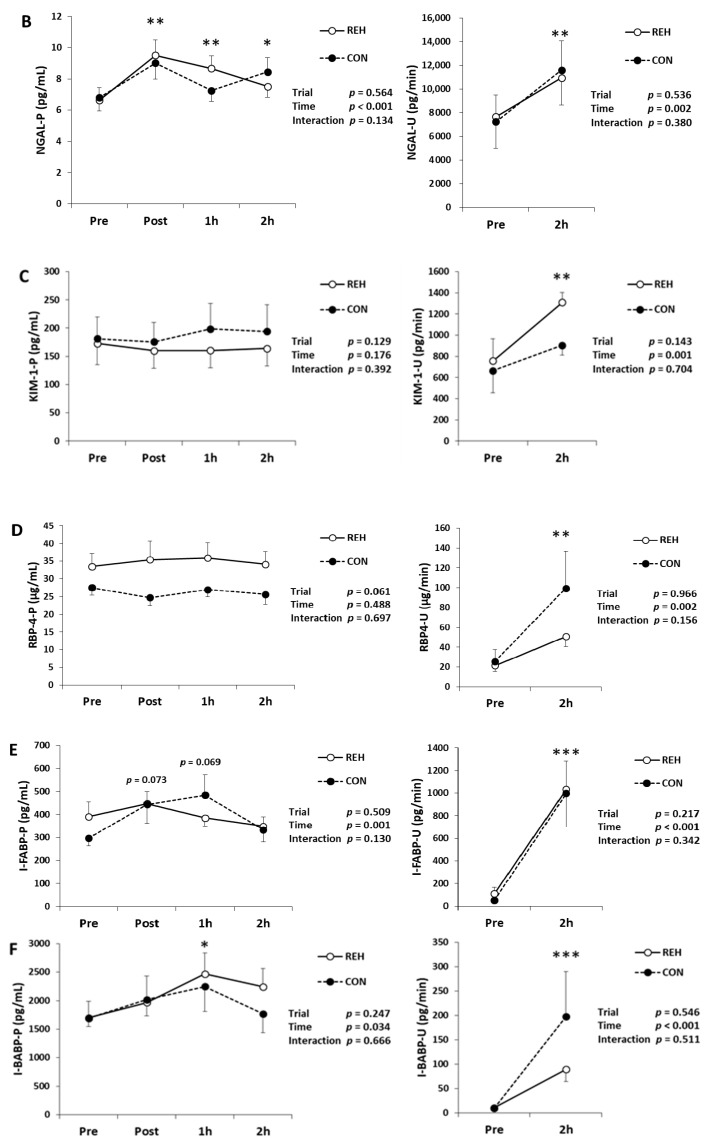

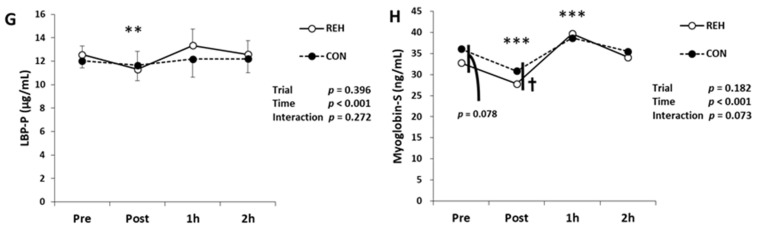
Changes in plasma and urinary (**A**) L-FABP, (**B**) NGAL, (**C**) KIM-1, (**D**) RBP-4, (**E**) I-FABP, (**F**) I-BABP, (**G**) LBP and (**H**) myoglobin. Values are presented as mean ± SE. * *p* < 0.05, ** *p* < 0.01, *** *p* < 0.001 vs. Pre. † *p* < 0.05 REH vs. CON in each time point. L-FABP, liver-fatty acid binding protein; NGAL, neutrophil gelatinase-associated lipocalin; KIM-1, kidney injury molecule 1; RBP-4, retinol binding protein 4; I-FABP, intestine-fatty acid binding protein; I-BABP, ileal-bile acid binding protein; LBP, lipopolysaccharide binding protein; REH, rehydrate trial; CON, control trial.

To evaluate renal damage/dysfunction, we measured BUN, creatinine, cystatin-C, L-FABP, NGAL, KIM-1 and RBP4 in blood and urine. Serum BUN and creatinine concentrations decreased immediately after exercise and increased 1 h after exercise (Table 1). Plasma cystatin-C concentration increased 1 h after exercise (Table 1). Urine protein, NAG and albumin concentrations increased after exercise (Table 1). Plasma L-FABP concentration increased 1 h and 2 h after exercise, as did urinary L-FABP concentration (Figure 2A). Plasma NGAL concentration increased immediately, 1 h and 2 h after exercise, and urinary NGAL concentration increased after exercise (Figure 2B). Plasma KIM-1 and RBP4 concentrations did not change after exercise, whereas urinary KIM-1 and RBP4 concentrations increased (Figure 2C,D). However, the changes to the renal damage/dysfunction markers did not differ between trials (interaction; *p* > 0.05).

To evaluate intestinal damage, we measured I-FABP and I-BABP in plasma and urine. A significant time effect was observed in plasma I-FABP concentrations, whereas I-FABP concentration trended to be higher at 1 h and 2 h compared with Pre (*p* = 0.073 and *p* = 0.069 respectively; Figure 2E). Urinary I-FABP concentration did increase after exercise (Figure 2E). Plasma I-BABP concentration increased 1 h after exercise, and urinary I-BABP concentration increased after exercise (Figure 2F). Plasma endotoxin concentration was under detection limit (Table 1). Plasma LBP concentration, an indirect marker of endotoxin exposure [26], decreased immediately after exercise (Figure 2G). However, the changes to the intestinal damage markers did not differ between trials (interaction; *p* > 0.05).

To evaluate muscle damage, we measured serum myoglobin concentration. Myoglobin decreased immediately after exercise and increased 1 h after exercise, and the myoglobin changes trended to differ between trials (interaction; *p* = 0.073, Figure 2H). Immediately after exercise, serum myoglobin concentration in CON was higher than that in REH. Pre exercise, serum myoglobin concentration in CON had a higher trend than that in REH (Figure 2H). These results indicate that beverage intake did not influence exercise-induced renal, intestinal and muscle damage, and this exercise mode did not induce endotoxemia.

### 3.3. Exercise-Induced Inflammation

**Figure 3:**
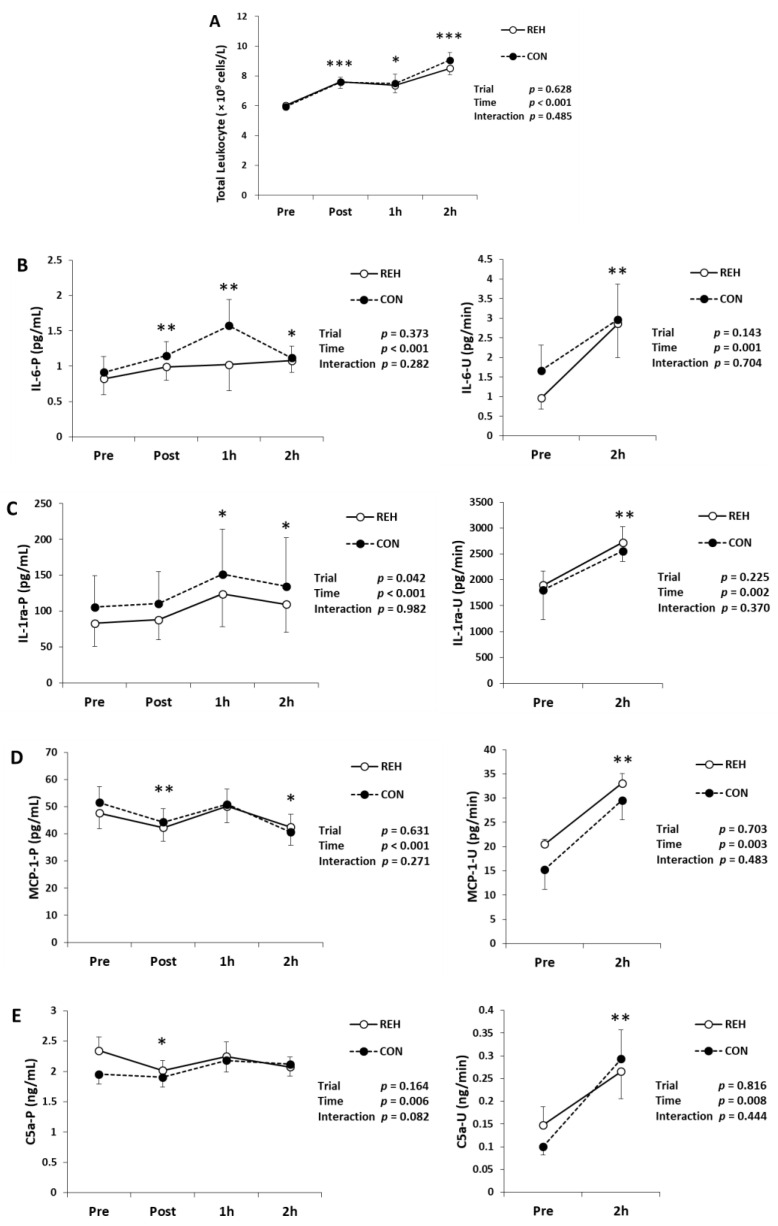

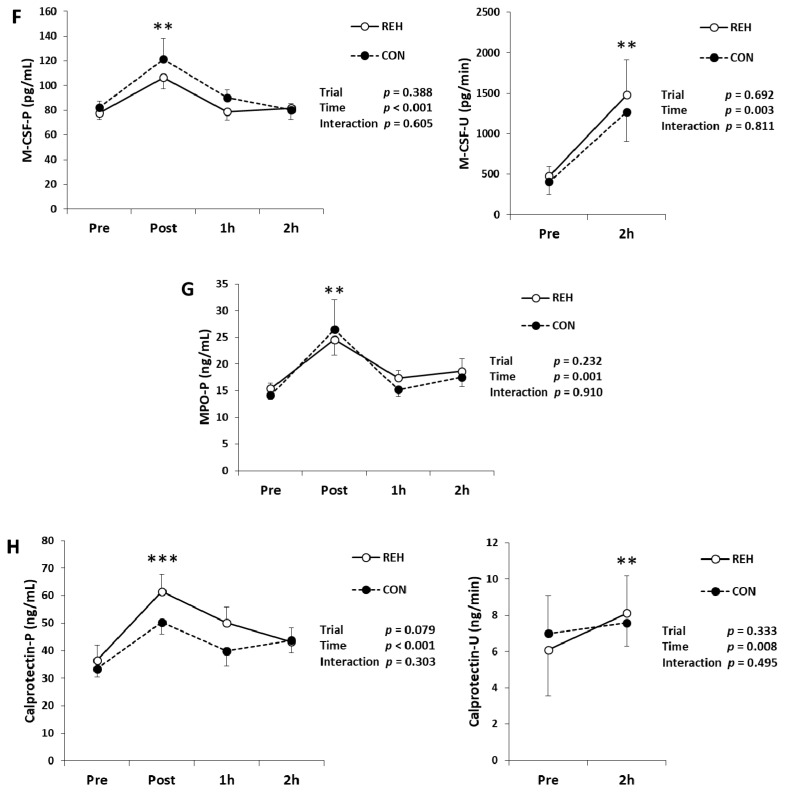
(**A**) Change in the number of total leukocytes in whole blood. Changes in (**B**) IL-6, (**C**) IL-1ra, (**D**) MCP-1, (**E**) C5a, (**F**) M-CSF, (**G**) MPO and (**H**) calprotectin in plasma (P) and urine (U). Values are presented as mean ± SE. * *p* < 0.05, ** *p* < 0.01, *** *p* < 0.001 vs. Pre. IL-6, interleukin-6; IL-1ra, interleukin-1 receptor antagonist; MCP-1, monocyte chemoattractant protein 1; C5a, complement 5a; M-CSF, macrophage colony-stimulating factor; MPO, myeloperoxidase; REH, rehydrate trial; CON, control trial.

To evaluate exercise-induced inflammation, we measured the total leukocytes in whole blood, TNF-α, IL-1ra, IL-2, IL-4, IL-6, IL-10, IL-18, IL-18BPa, C5a, MCP-1, G-CSF, M-CSF, MPO and calprotectin in plasma and urine. The total leukocytes increased immediately, 1 h and 2 h after exercise (Figure 3A). Plasma IL-6 concentration increased immediately, 1 h and 2 h after exercise, and urinary IL-6 concentration also increased after exercise (Figure 3B). Plasma IL-1ra concentration increased 1 h and 2 h after exercise and urinary IL-1ra concentration also increased after exercise (Figure 3C). Plasma MCP-1 concentration decreased immediately and 2 h after exercise. However, urinary MCP-1 concentration increased after exercise (Figure 3D). Plasma C5a concentration decreased immediately after exercise. However, urinary C5a concentration increased after exercise (Figure 3E). Plasma M-CSF, MPO and calprotectin concentrations increased immediately after exercise. Urinary M-CSF and calprotectin concentrations increased after exercise, whereas urinary MPO concentration was under the detection limit (Figure 3F–H). Plasma and urinary IL-10 and IL-18BPa concentrations did not change after exercise (Table 2). Plasma G-CSF concentration did not change after exercise and urinary G-CSF concentration was under the detection limit (Table 2). Plasma and urinary TNF-α, IL-2, IL-4 and IL-18 concentrations were under the detection limit (Table 2). The changes in the above inflammatory parameters did not differ between trials (interaction; *p* > 0.05). These results indicate that beverage intake did not influence exercise-induced inflammation.

### 3.4. Exercise-Induced Oxidative Stress and Antioxidant Substances

**Figure 4:**
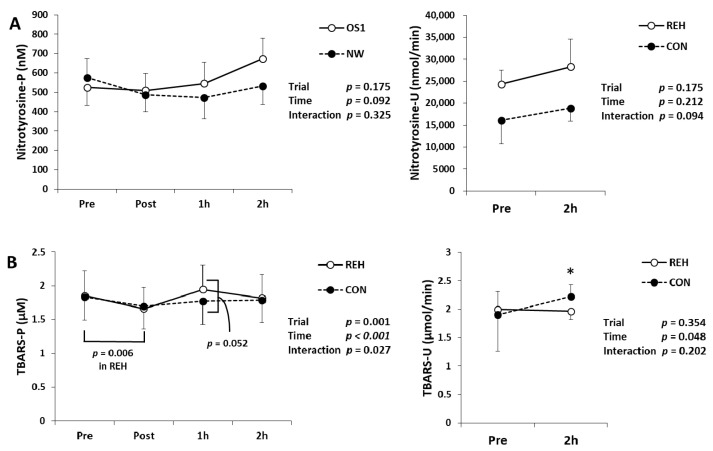

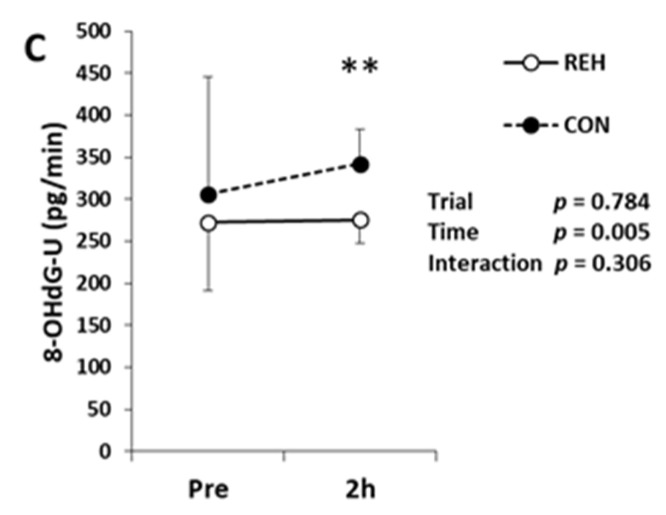
Changes in (**A**) nitrotyrosine, (**B**) TBARS and (**C**) 8-OHdG in plasma (P) and urine (U). Values are presented as mean ± SE. * *p* < 0.05, ** *p* < 0.01 vs. Pre. TBARS, thiobarbituric acid reactive substances; 8-OHdG, 8-hydroxy-2′-deoxyguanosine; REH, rehydrate trial; CON, control trial.

We measured d-ROMs, BAP, uric acid, nitrotyrosine and TBARS in serum, plasma and urine to evaluate oxidative stress and antioxidant substances. Serum d-ROMs and BAP concentrations did not change after exercise (Table 3). Serum uric acid concentration decreased immediately after exercise and increased 1 h and 2 h after exercise (Table 3). Urinary uric acid concentration increased after exercise (Table 3). Plasma and urinary nitrotyrosine concentrations did not change after exercise (Figure 4A). Plasma TBARS concentration showed different changes between trials (interaction; *p* = 0.027). Immediately after exercise, plasma TBARS concentration in REH decreased compared to Pre. One hour after exercise, plasma TBARS concentration in REH had a higher trend than that in CON (*p* = 0.052) (Figure 4B). Urinary TBARS and 8-OHdG concentrations increased after exercise (Figure 4B,C). Except for plasma TBARS, the changes of the oxidative stress markers did not differ between trials (interaction; *p* > 0.05). These results indicate that beverage intake quickly restored oxidative stress concentration in plasma.

### 3.5. Hormones, Glucose and FFA

**Figure 5:**
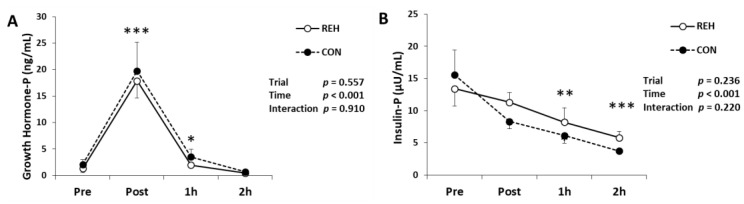

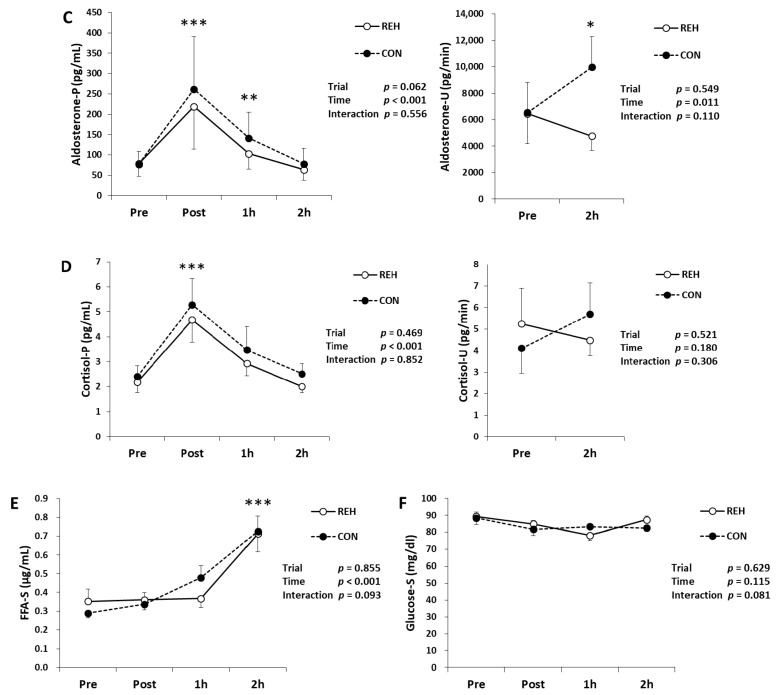
Changes in (**A**) growth hormone, (**B**) insulin, (**C**) aldosterone, (**D**) cortisol, (**E**) FFA and (**F**) glucose in plasma (P), serum (S) and urine (U). Values are presented as mean ± SE. * *p* < 0.05, ** *p* < 0.01, *** *p* < 0.001 vs. Pre. FFA, free fatty acid; REH, rehydrate trial; CON, control trial.

We measured hormones to evaluate the stress response. Plasma growth hormone concentration increased immediately and 1 h after exercise (Figure 5A). Plasma insulin concentration decreased 1 h and 2 h after exercise (Figure 5B). Plasma aldosterone concentration increased immediately after exercise and urinary aldosterone concentration did not change (Figure 5C). Plasma cortisol concentration increased immediately after exercise, whereas urinary cortisol concentration did not change (Figure 5D). Serum FFA concentration increased 2 h after exercise (Figure 5E). Serum glucose concentration did not change after exercise (Figure 5F). The changes in the above parameters did not differ between trials (interaction; *p* > 0.05). These results indicate that beverage intake did not affect hormonal responses.

## 4. Discussion

In the present study, plasma TBARS concentration showed different changes between trials. Furthermore, plasma TBARS concentration in REH decreased immediately after exercise and tended to be higher than CON 1 h after exercise. This result suggests that rehydration restores the decreased oxidative stress by exercise more quickly. One possible interpretation is that rehydration may promote exercise adaptation because exercise-induced oxidative stress contributes to exercise adaptation [27]. In contrast to TBARS, other oxidative stress markers and antioxidants, d-ROMS, nitrotyrosine, 8-OHdG, BAP and uric acid were not influenced by beverage intake. These differences may be due to the differences in the oxidized metabolites detected. However, we could not elucidate the mechanisms of this phenomenon. Endurance exercise induces oxidative stress in various organs such as muscle and liver [1,13,14,15]. As a source of oxidative stress, it may be important to investigate the effects of rehydration on oxidative stress in each organ.

In the present study, the biomarkers of inflammation and organ damage increased following exercise. However, the changes in these biomarkers did not differ between trials. These results suggest that beverage intake after exercise for rehydration does not prevent organ damage and inflammation. There are two possible reasons for the above results. One reason is the amount of ingested carbohydrates (CHO). A lot of studies have reported that CHO-containing drinks attenuate endurance exercise-induced inflammation [16,17] and muscle and intestinal damage [18,19,20]. In the present study, we used beverages containing some CHO. However, plasma glucose did not increase in the REH trial. Therefore, the amount of ingested CHO may be too small to attenuate exercise-induced organ damage and inflammation. Another reason for the results achieved is the timing of the beverage intake. Water, isotonic sports drink or CHO-containing beverage intake before and during exercise attenuates endurance exercise-induced muscle and intestinal damage and inflammation [16,17,18,19,20,21,22]. However, CHO-containing beverage intake after endurance exercise did not attenuate muscle damage and inflammation [28]. Similarly, the research of Tanisawa et al. [28] reported that beverage intake after exercise for rehydration did not attenuate exercise-induced muscle damage and inflammation. However, several studies have reported that CHO-containing beverage intake after exercise augments eccentric exercise-induced inflammation [29,30,31]. Therefore, the effects of beverage intake after exercise on exercise-induced organ damage and inflammation may depend on the exercise mode. Considering these studies and our results, beverage intake before and during exercise may effectively prevent endurance exercise-induced organ damage and inflammation.

In the present study, the plasma endotoxin concentration was undetermined. However, plasma LBP concentration, an indirect marker of endotoxin exposure [26], did not increase. These results suggest that our exercise protocol did not increase plasma endotoxin concentration, which is consistent with a previous review which found that exercise undertaken for less than 2 h does not increase plasma endotoxin concentration [26]. Intestinal permeability is an important factor for exercise-induced endotoxemia [1,5]. However, 1 h of exercise induces intestinal hyperpermeability and damage but does not result in endotoxemia [32]. Furthermore, high-intensity interval training (HIIT) induces intestinal hyperpermeability and damage [33]. These results suggest that exercise lasting less than 2 h does not induce endotoxemia but may induce intestinal hyperpermeability. Gut-derived endotoxins in the portal vein are removed by phagocytosis of Kupffer cells, and acute exercise increases the phagocytotic ability of these cells [34]. Therefore, if endotoxin leakage in the gut is within the phagocytotic ability of Kupffer cells, endotoxins in the portal vein may not enter into systemic circulation. In the present study, we hypothesized that exercise induces inflammation via gut-derived endotoxins. However, our results suggest that after short-duration exercise, factors other than endotoxins trigger exercise-induced inflammation. Organ damage itself and stress hormones (e.g., cortisol, catecholamines and growth hormone) are the other triggers of exercise-induced inflammation [1,9,35]. In the present study, organ damage and stress hormones may induce inflammation independently of gut-derived endotoxins. Because beverage intake did not influence organ damage and stress hormones in the present study, beverage intake may not influence exercise-induced inflammation.

## 5. Limitation

This study was limited to men to avoid the effects of menstrual cycles on exercise-induced inflammation [36,37]. Therefore, the results of this study cannot be generalized because the effects of post-exercise beverage intake may be different in women.

In this study, we measured many cytokines in plasma and urine. However, several parameters were under the detection limit. Therefore, we could not evaluate the effects of post-exercise rehydration on several cytokines. Generally, cytokine concentrations in body fluids are very low, and some cases are expected to be under the detection limit in healthy subjects [8,38]. Therefore, it is necessary to identify such low-concentration substances to assess physiological stress to exercise using more sensitive assays.

## 6. Conclusions

Beverage intake after exercise to recover from dehydration did not attenuate exercise-induced organ damage, inflammation and oxidative stress in healthy males, and exercise induced inflammation independently of gut-derived endotoxins. However, rehydration restored exercise-induced oxidative stress more quickly.

## Figures and Tables

**Table 1:** Changes in organ damage markers in serum (S)/plasma (P) and urine (U).

	Unit	Trial	Pre	Post	1h	2h	Trial	Time	Interaction
BUN-S	mg/dL			**			0.907	<0.001	0.902
		REH	18.88 ± 1.05	17.76 ± 1.12	19.80 ± 1.12	18.90 ± 1.16			
		CON	19.02 ± 1.23	17.67 ± 1.12	20.05 ± 1.47	19.13 ± 1.36			
Creatinine-S	mg/dL			**	**		0.429	<0.001	0.105
		REH	0.92 ± 0.03	0.84 ± 0.03	0.98 ± 0.03	0.92 ± 0.03			
		CON	0.95 ± 0.04	0.88 ± 0.03	0.98 ± 0.03	0.93 ±0.04			
Cystatin C-P	mg/mL				***		0.232	<0.001	0.326
		REH	0.74 ± 0.01	0.72 ± 0.01	0.80 ± 0.01	0.76 ± 0.02			
		CON	0.74 ± 0.01	0.71 ± 0.02	0.77 ± 0.02	0.73 ± 0.02			
Urine Protein-U	µg/min					**	0.528	0.001	0.183
		REH	41.08 ±10.48			73.23 ± 6.93			
		CON	26.31 ± 5.84			75.10 ± 8.93			
NAG-U	mU/min					**	0.930	0.001	0.624
		REH	29.88 ± 7.05			54.77 ± 6.13			
		CON	26.52 ± 7.26			56.82 ± 6.68			
Alubmin-U	mU/min					***	0.609	<0.001	0.469
		REH	43.82 ± 11.29			338.26 ± 67.74			
		CON	47.80 ± 12.31			386.24 ± 74.32			
Endotoxin-P	IU/mL	REH	ND	ND	ND	ND			
		CON	ND	ND	ND	ND			

Values are presented as mean ± SE. ** *p* < 0.01, *** *p* < 0.001 vs. Pre. BUN, blood urea nitrogen; NAG, *N*-acetyl-β-d-glucosaminidase; ND, not detected; REH, rehydrate trial; CON, control trial.

**Table 2:** Changes in the biomarkers of inflammation in plasma (P) and urine (U).

	Unit	Trial	Pre	Post	1h	2h	Trial	Time	Interaction
TNF-α-P	pg/mL	REH	ND	ND	ND	ND			
		CON	ND	ND	ND	ND			
TNF-α-U	pg/mL	REH	ND			ND			
		CON	ND			ND			
IL-2-P	pg/mL	REH	ND	ND	ND	ND			
		CON	ND	ND	ND	ND			
IL-2-U	pg/mL	REH	ND			ND			
		CON	ND			ND			
IL-4-P	pg/mL	REH	ND	ND	ND	ND			
		CON	ND	ND	ND	ND			
IL-4-U	pg/mL	REH	ND			ND			
		CON	ND			ND			
IL-10-P	pg/mL	REH	5.96 ± 1.00	5.61 ± 0.89	5.78 ± 0.64	4.64 ± 0.66	0.540	0.091	0.655
		CON	5.34 ± 0.73	5.74 ± 1.07	6.61 ± 0.91	5.61 ± 0.98			
IL-10-U	pg/min	REH	9.85 ± 1.33			13.83 ± 1.19	0.006	0.214	0.707
		CON	7.98 ± 2.18			7.84 ± 0.83			
IL-18-P	pg/mL	REH	ND	ND	ND	ND			
		CON	ND	ND	ND	ND			
IL-18-U	pg/mL	REH	ND			ND			
		CON	ND			ND			
IL-18BPa-P	ng/mL	REH	79.53 ± 11.01	79.56 ± 11.55	66.39 ± 12.52	75.52 ± 11.94	0.904	0.146	0.683
		CON	74.10 ± 10.17	81.61 ± 11.93	70.10 ± 9.19	72.63 ± 5.25			
IL-18BPa-U	ng/min	REH	94.55 ± 21.41			133.49 ± 30.00	0.025	0.287	0.838
		CON	62.17 ± 24.42			52.75 ± 11.45			
G-CSF-P	pg/mL	REH	14.17 ± 2.62	12.69 ± 2.30	14.41 ± 2.32	13.16 ± 2.23	0.789	0.292	0.246
		CON	13.49 ± 2.53	12.32 ± 2.01	15.37 ± 2.15	13.36 ± 2.08			
G-CSF-U	pg/mL	REH	ND			ND			
		CON	ND			ND			
MPO-U	ng/mL	REH	ND			ND			
		CON	ND			ND			

Values are presented as mean ± SE. IL, interleukin; IL-18BPa, IL-18 binding protein α; G-CSF, granulocyte colony-stimulating factor; MPO, myeloperoxidase; ND, not detected; REH, rehydrate trial; CON, control trial.

**Table 3:** Changes in oxidative stress and antioxidant substances in serum (S) and urine (U).

	Unit	Trial	Pre	Post	1h	2h	Trial	Time	Interaction
d-ROMs-S	U-CARR	REH	237.77 ± 10.67	234.38 ± 10.36	237.37 ± 10.64	233.00 ± 11.37	0.138	0.327	0.567
		CON	230.00 ± 9.50	218.87 ± 9.87	224.70 ± 10.90	227.84 ± 10.73			
BAP-S	mmol/L	REH	2.28 ± 0.05	2.32 ± 0.05	2.31 ± 0.07	2.36 ± 0.06	0.218	0.343	0.474
		CON	2.20 ± 0.05	2.26 ± 0.04	2.32 ± 0.06	2.23 ± 0.03			
Uric Acid-S	mg/dL			***	***	**	0.427	<0.001	0.602
		REH	5.43 ± 0.35	5.11 ± 0.34	7.01 ± 0.58	6.52 ± 0.52			
		CON	5.76 ± 0.29	5.26 ± 0.26	7.04 ± 0.35	6.65 ± 0.37			
Uric Acid-U	µg/min					*	0.229	0.025	0.994
		REH	564.32 ± 86.24			775.35 ± 80.89			
		CON	445.87 ± 19.81			658.69 ± 68.15			

Values are presented as mean ± SE. * *p* < 0.05, ** *p* < 0.01, *** *p* < 0.001 vs. Pre. d-ROMS, derivatives of reactive oxygen metabolites; BAP, biological antioxidant potential; REH, rehydrate trial; CON, control trial.

## Data Availability

The raw data presented in this study are available upon request from the corresponding author. The raw data are not publicly available.
